# Surgical management of vascular malformations of the upper extremity: A review of current literature

**DOI:** 10.1016/j.jpra.2022.05.008

**Published:** 2022-05-21

**Authors:** Margriet H.M. van Doesburg, Houda Harbech, Max M. Lokhorst, Corstiaan C. Breugem

**Affiliations:** Department of Plastic, Reconstructive and Hand Surgery. Amsterdam University Medical Centre, Meibergdreef 9, 1105 AZ Amsterdam

**Keywords:** Vascular malformations, Upper extremity, Surgery, Recurrence, Complications

## Abstract

**Introduction:**

Vascular malformations of the upper extremity are uncommon, and there is great heterogeneity in their occurrence and appearance. There is no golden standard for the treatment of vascular malformations of the upper extremity and limited evidence on this subject has been published.

**Objective:**

This review aims to answer the question whether surgical treatment leads to less recurrence and complications than non-surgical treatment for patients with vascular malformations of the upper extremity.

**Materials and methods:**

A literature search in PubMed was performed up to September 2019 by using the following terms: vascular malformation, upper extremity and surgery. Inclusion criteria were: a mean follow-up duration of at least 12 months [1], outcome measurements including recurrences and/or complications [2] and the involvement of patients with vascular malformations of the upper extremity [3].

**Results:**

In total, 883 articles were found, of which seven were included in this review. A total of 358 patients were included in these studies, including 208 patients with upper extremity vascular malformations. Minor surgical complications were seen in 20% of the cases, and major complications occurred in 6%. Recurrence was reported in 32% of the cases.

**Conclusion:**

Surgery for vascular malformations of the upper extremity can be a safe and effective treatment option, although some cases are better off when treated non-surgically. Literature shows various complication rates for non-surgical treatment of upper extremity vascular malformations. To determine in which case surgery is the better option, we should identify factors leading to surgical complications.

## Introduction

Vascular malformations are congenital abnormally developed vessels, often dilated and tortuous. These malformations are a subset of a larger group of vascular anomalies and should not be mistaken for hemangiomas, which show rapid growth and development, in contrast to the slowly growing vascular malformations.^1^ Vascular malformations are hard to treat and can sometimes be life-threatening.^2^ According to the International Society for the Study of Vascular Anomalies (ISSVA), the malformations are categorized as low or high flow.^26^ Low-flow malformations can be classified into their primary cell type: venous, lymphatic, or capillary, while high-flow malformations are of the arteriovenous type.^3^ Symptoms of vascular malformations are diverse and depend on the type of malformation, but can lead to pain, thrombosis, compression of functional structures and cosmetic complaints.

Treatment of vascular malformations generally requires a multidisciplinary approach, usually consisting of at least a dermatologist, interventional radiologist, plastic surgeon and a vascular surgeon. The first step in vascular malformation management is often conservative therapy.^4^ Further treatment is based on histology of the lesion, available expertise and the patient's preferred treatment option.^5^ Surgery and interventional radiology play an essential role and can be used combined.^4^ Non-surgical treatment options include embolization, sclerotherapy and laser therapy. Additionally, recent advancements in genetics have allowed for the development of targeted therapies, which are currently being studied and used off-label. These therapies might prove beneficial especially for patients with therapy-resistant and diffuse lesions.[CB1]

Management of upper extremity malformations can be a surgical challenge, especially when it comes to lesions of the hand and wrist. The main reason for this complicated surgical management is the need to maintain upper extremity function.^2^ Other factors causing obstacles in the treatment are their diffuse localization and concerns regarding peripheral blood supply related to treatment options.^6^

Different surgical techniques can be used to eradicate vascular malformations. Previous literature on arteriovenous malformations (AVMs) suggests that curation of malformations can be accomplished after complete surgical removal^7^^,^^8^ or destruction of the nidus.^8^^,^^9^ Clearly defined malformations are suitable for surgical treatment. Complete removal is not desired in some cases, for example, in patients suffering from life- or limb-threatening complications of AVMs. These patients may profit from debulking surgery. This surgical technique removes portions of the malformation, without necessarily forming recurrent lesions.^10^ Even though this technique is controversial and considered a major operation with noteworthy risks of complications, it could relieve severe symptoms.^11^, ^12^, ^13^, ^14^, ^15^ Dated evidence suggests that diffuse malformations are residue tissue from embryonic development and have features of mesenchymal cells, which results in re-growth of these lesions after debulking surgery.^10^

In some patients, compartmentalization is a different option for surgical management. In this technique, the surgeon places big sutures in numerous locations of the malformation to create multiple divisions within the lesion.^16^ Outcomes on recurrence after this surgery are inadequately reported.^14^

Non-surgical treatment of vascular malformations, such as embolization, has developed progressively over time. Embolization of AVMs can minimize flow. This outcome could be short-term or used in a palliative setting.^8^^,^^17^, ^18^, ^19^ Sclerotherapy is acknowledged as an individual treatment for vascular malformations.^8^ For venous malformations (VMs), poor results of embolization have been shown, but sclerotherapy could be considered effective for these malformations.^20^ Repeated courses of sclerotherapy can clinically improve VMs^21^ and this technique can also be used as an adjuvant therapy to surgery. Studies show that preoperative embolization reduces haemorrhage during surgery.^7^^,^^9^

There is no golden standard for the treatment of vascular malformations of the upper extremity and limited evidence is available on this subject. This study was designed to review the literature on surgical treatment for vascular malformations of the upper extremity. The aim of this review was to investigate whether surgical treatment of upper extremity vascular malformations leads to less complications and recurrence than non-surgical treatment.

## Materials and methods

We followed the guidelines of the Preferred Reporting Items for Systematic Reviews and Meta-Analyses (PRISMA) statement for this systematic review. The review was not registered.

### Identification of relevant articles

A literature search in PubMed was performed on March 19, 2020, using the following terms: vascular malformation, upper extremity and surgery. The detailed search is shown in Table 1. This search was created with the help of a clinical librarian. The reference lists of included articles were screened on relevant studies that did not appear in the PubMed search.

**Table 1:** PubMed search

	**Synonyms**

Vascular malformation	vascular malformation* [tiab] OR vascular anomaly [tiab] OR vascular anomalies [tiab] OR AVM [tiab] OR arteriovenous malformation* [tiab]
	**AND**
Upper extremity	upper extremity [tiab] OR upper limb [tiab] OR upper limbs [tiab] OR brachial [tiab] OR brachi [tiab] OR arm [tiab] OR arms [tiab] OR hand [tiab] OR hands [tiab] OR wrist [tiab] OR wrists [tiab]OR finger [tiab] OR fingers [tiab] OR phalanx [tiab] OR phalanges [tiab] OR metacarpus [tiab] OR metacarpi [tiab] OR metacarpal [tiab] OR palm [tiab] OR palms [tiab]
	**AND**
Surgery	surgery [tiab] OR surgical treatment [tiab] OR surgical excision [tiab] OR surgical removal [tiab]

### Selection of relevant articles

All articles that were retrieved from the PubMed search were screened on title and abstract by one reviewer. Criteria for inclusion and exclusion are shown in Table 2. Studies reporting surgical treatment of vascular malformations (AVM, VM, lymphatic malformation (LM) and capillary malformation (CM)) of the upper extremity in at least 10 patients were included. Studies that also reported vascular malformations of other regions, were only included if data were reported separately for the upper extremity.

**Table 2:** In- and exclusion criteria

Inclusion criteria	Exclusion criteria

- All types of vascular malformations of the upper extremity - Patients were treated by surgery - Original study (observational study or randomized controlled trial) - Outcomes reported of at least 10 patients - Mean follow-up ≥ 12 months - Complications or recurrence reported as outcome measure	x Articles in other languages than Dutch or English x Non-human studies x Vascular malformations located in the central nervous system or purely the visceral organs x Case reports x Outcomes not reported separately for upper extremity

### Critical appraisal

To assess the methodological quality of the studies, the Grading of Recommendations, Assessment, Development and Evaluations (GRADE) approach was used.^27^

### Data extraction

Data on study design, number of participants, type and lesion localization, treatment, outcomes and follow-up duration were collected. Relevant interventions for this review were: surgery and non-surgical treatment (embolotherapy, sclerotherapy and laser treatment). Relevant outcomes were complications and recurrence.

### Data analysis

Recurrence and complication rates were calculated per patient, including calculation of a weighted mean. Numbers of patients who received surgery, cases of minor and major complications and recurrence were manually collected for each study. An average complication and recurrence rate was calculated, weighted the complication and recurrence numbers were averaged and weighted on the basis of the number of patients included.

## Results

### Selection of studies

**Figure 1:**
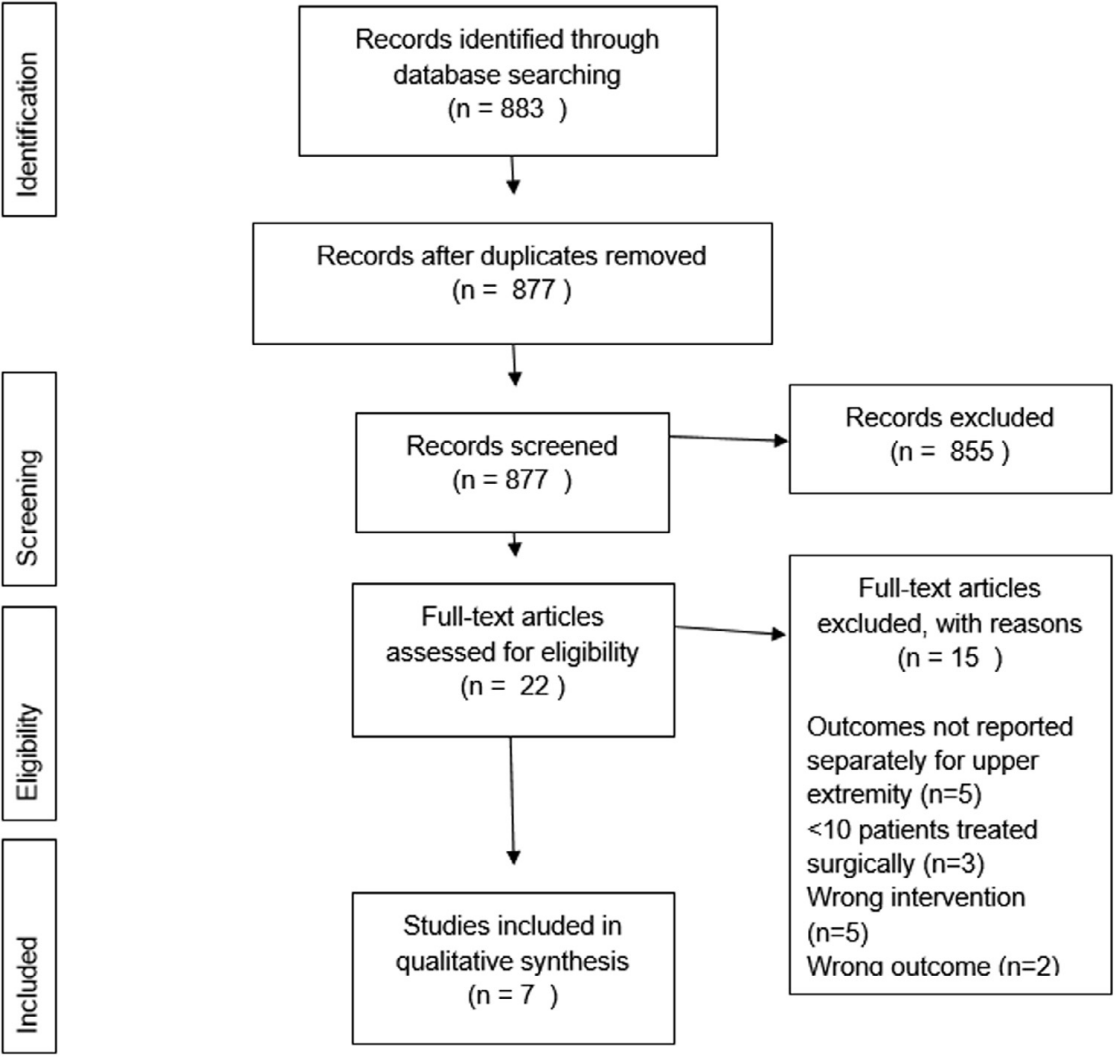
PRISMA flow diagram of literature search

The PubMed search, shown in Table 1, and manual screening yielded 883 studies of which seven were included with a total of 358 patients (Figure 1).^2^^,^^6^^,^^17^^,^^19^^,^^22^^,^^23^ Aside from patients with vascular malformations of the upper extremity, several studies also included patients with vascular malformations of other body regions. These were excluded if their results were not reported per region (n=5). Other reasons for exclusion after full-text assessment were <10 patients treated surgically (n=3) and wrong intervention (n=5) or outcome (n=2).

### Study and patient characteristics

Study type, patient and lesion characteristics and quality assessment of each study are reported in Table 3. Lesion characteristics were location, size, tissue involvement and symptoms prior to treatment. Treatment characteristics, including perioperative treatment, surgical treatment, outcome measures, outcome measurement instruments and complications are shown in Table 4.

**Table 3:** Study, patient and lesion characteristics. AVM = arteriovenous malformation, VM = venous malformation, LM = lymphatic malformation, NR = not reported, *not reported separately for surgically treated group, **not reported separately for the patients of which the outcomes were presented.

Author, year, country	Study type	No. of patients (age)	Localization of vascular malformation	Size of vascular malformation	Tissue involvement	Symptoms prior to treatment	Prior treatment	Quality assessment
Upton, 1999, USA	Retrospective	270 (1–56) *VM: 125* *LM: 47* *CM: 32* *AVM: 33* *Combined: 33*	Upper limb	NR	NR	*LM/VM/CM* Swelling Cutaneous discoloration Overgrowth of the limb Recurrent infections Pain/paresthesias Functional impairment *AVM* Swelling Pain with exercise Hyperhydrosis Diastal ischemia Increased warmth of the limb Thrill and pulsations Compression neuropathies Progressive pain	NR	Very low
Mendel, 1997, USA	Retrospective	17 (5–87) *AVM: 6* *VM: 2* *Hemangioma:6* *Lymphangioma: 3*	Hand: 5 Finger: 5 Arm: 5 Elbow: 1 Wrist: 1	Diffuse: 10 Local: 7	NR	Painful or enlarging mass Decreased range of motion Neurologic symptoms	None	Very low
Hill, 1993, Singapore	Retrospective	15 (3-37) *Low flow: 9 (60%)* *High flow: 6 (40%)*	Hand: 9 (60%) Forearm (and hand): 5 (33%) Elbow: 1 (7%)	Diffuse: 10 Local: 7	Skin and subcutaneous: 9 Muscle/tendon: 14 Bone: 5 Nerve: 5	Pain: 11 (73%) Progressive swelling: 13 (87%) Impairment of function: 5 (33%)	Surgery: 4 Radiotherapy: 1	Very low
**AVM**								
White, 2000, USA	Retrospective	20 (13–63)	Upper extremity: 11 Lower extremity: 9	NR	NR	Pain: 10 Ulceration: 1 Dysfunction of the hand: 7 Bony erosion: 2 Bleeding: 2	Skeletization: 3 Embolization: 1	Very low
**VM**								
Al-Qattan^28^, Saudi Arabia	Retrospective	15 (19–50)	Hand	Few mm – 3cm	Subcutaneous	Swelling: 15 (100%) Pain: - ‘mild throbbing’: 6 (40%) - ‘dull aching’: 5 (33%) Triad of pain, tenderness and cold intolerance: 2 (13%) History of minor bleeding following trauma: 1 (7%) Ridging of the nail: 1(7%)	NR	Very low
Enjolras, 1997, France	Retrospective	27 (0–28)	Upper limb: 11 (41%) Lower limb: 16 (59%)	NR	Bone/joint involvement: 9	Blue discoloration of the skin: 100% Pain: 100% Elbow pain and limited motility of this joint: 4 (	Compression: all	Very low
Mendonca, 2010, United Kingdom	Retrospective	33 (0–18)	Upper limb: 19 Lower limb: 14	NR	Skin/subcutaneous: 11 Fascia/muscle involvement: 4 Bone/joint involvement: 3 Diffused whole limb involvement: 1	Pain Swelling Ulceration Bleeding Loss of function	None	Very low

**Table 4:** Treatment characteristics, outcome measures and corresponding results (including complications)

Author, year, country	No. of patients* (age)	Perioperative treatment	Surgical treatment	Control group	Outcome measures and results	Outcome measurement instruments	Complications	Follow-up in months, mean (range)
Upton, 1999, USA	270 (1-56)	*Preoperative* Embolization: 1 *Postoperative* Release of contractures: 12 Scar revision: 5 Tissue expansion and local flap transvers: 6 Excision and rerouting of neuromas: 3	Subtotal resection: Total resection: 141	Flash lamp-pumped pulsed dye laser: 3 Sclerotherapy (ethanol or STD): 9 Embolization: 2	Number of patients/number of operative procedures: *CM: 0* *VM: 53/76* *LM: 38/63* *AVM: 24/58* *Combined:26/63*	NR	Minor: 53 Major: 16 *Slow-flow 22%* *Fast-flow 28%*	204 (12-300)
Mendel, 1997, USA	17 (5–83)	NR	Resection: 12 Primary amputation: 2	Embolization (metrizamide): 1	Recurrence: 71%	Clinical notes, pathology reports, surgery notes, radiologic studies	Minor: 0 Major: 0	144 (4–432)
Hill, 1993, Singapore	15 (3–37)	Postoperative embolization: 1	Total resection: 7 (47%) Subtotal resection: 8 (53%)	None	Recurrence: 47% Time interval for recurrence: 2-18 months	NR	Minor: 0 Major: 2 (13%)	12–180
**AVM**								
White, 2000, USA	11 (13-63)	Preoperative embolization (cyanoacrylate): all	Total resection: 4 Primary amputation: 2	Only embolization: 5	Recurrence: 0 Complete bone regrowth: 1 Complete relief of arm swelling: 1 Asymptomatic: 7 Marked improvement:1 Lost to follow-up after 1 year: 2	Recorded reviews	Minor: 1 Major: 0	88 (12–214)
**VM**								
^28^, Saudi Arabia	15 (19–50)	NR	Total resection: 15	None	Recurrence: 0	NR	NR	12–60
Enjolras, 1997, France	11 (0–28)	Preoperative skin expansion: 1	Total resection: 5 Subtotal resection: 1	Sclerotherapy: 2 No treatment reported: 3	*Surgical treatment* Improved: 2 (33%) Mediocre: 1 (17%) Unchanged: 2 (33%) Bad: 1 (17%) *Sclerotherapy* Mediocre: 1 (50%) slightly improved: 1 (50%)	NR	Minor: 1 Major: 0	108 (12–204)
Mendonca, 2010, United Kingdom	19 (0–18)	Sclerotherapy	Subtotal resection: 11	Sclerotherapy (STD)	*Surgical treatment* Symptoms improved: all Recurrence: 3 (27%) *Sclerotherapy* Symptoms improved: all	Clinical photographs and recorded reviews of follow-up clinic	*Surgical treatment* Minor: 2 Major: 0 *Sclerotherapy* Minor: 2 (0-67%) Major: 1 (0-33%)	29 (7–84)

M, malformation; V, venous; L, lymphatic; C, capillary; A, arterial; STD, sodium tetradecylsulphate

⁎ number of patients with upper extremity vascular malformation

### Complications

Complication rates for each study and total complication rates are shown in Table 5. We divided them into minor and major complications. Major complications require further invasive treatment. Every patient can have more than one complication.

**Table 5:** Total recurrence and complication rates

No. patients (nST*)	Minor complications (%)	Major complications (%)	Recurrence
270 (141)	53 (38%)	16 (11%)	NR
17 (14)	0	0	71%
15 (15)	0	2 (13%)	41%
11 (6)	1 (9%)	0	0%
15 (15)	NR	NR	0%
11 (6)	0	1 (9%)	NR
19 (11)	2 (18%)	0	NR
Total: 358 (208)	56	19	–
Weighted mean	**38.9 (20%)**	**11.9 (6%)**	**32%**

⁎ nST, number of surgically treated patients

A total of 358 patients are included in this review. Surgery was performed in 208 patients (46%). Surgical complication rates were, respectively, 20% and 6% for minor and major complications.

Minor complications were seen in 20% (weighted mean) of all patients, ranging from 0% to 38%. Upton et al^2^ reported cases of seroma/hematoma, skin loss, vesicle formation, wound dehiscence, neuroma, compartment syndrome, hypertrophic scar, cellulitis and persistent oedema. Enjolras et al^17^ showed one case of severe scarring after surgery. Mendonca et al^6^ reported one case of wound dehiscence and a case of skin necrosis.

Major complications occurred in 6% of the reported cases. Hill et al^22^ reported two cases of gangrene after incomplete excision. Major complications reported in Upton et al^2^ included reflex sympathetic dystrophy and amputation.

### Recurrence

Recurrence rates are shown in Table 5. Recurrence was reported as an outcome measurement in four out of seven studies. Recurrence after surgical treatment was seen in 32%. Upton et al^2^ observed recurrences 1 year post surgery. Almost all type 3 lesions in Mendonca et al^6^ recurred. In the study of Hill et al.^22^, 71% of the recurrence cases were of the diffuse type.

## Discussion

To date, there are no standard treatments for vascular malformations of the upper extremity, a rare and therapeutically challenging condition. The location of these malformations makes surgical treatment difficult, due to potential loss of function, especially in the hand and wrist.

The aim of this review was to investigate whether surgical treatment leads to less recurrence and complications as compared to non-surgical treatment for patients with vascular malformation of the upper extremity.

Based on a total of 358 cases, studies included in this review tend to show a minor complication rate of 20% and a 6% major complication rate in surgically treated patients. This indicates that surgery for vascular malformations of the upper extremity can be a safe treatment option, although some cases are better off when treated non-surgically. Literature shows various complication rates for non-surgical treatment of upper extremity vascular malformations. To determine in which case surgery is the better option, we should identify factors leading to surgical complications.

### Recurrence

To measure the effect of surgery in upper extremity vascular malformations, we used recurrence as an outcome measure. Based on three case series, we found a recurrence rate of 32%, while no evidence was found on the effect of incomplete excision on recurrence, compared to complete resection. The term recurrence should be clarified, as some lesions are impossible to completely eradicate. In these cases, debulking surgery is performed to relieve symptoms and recurrence is defined as recurrence of symptoms.

A remarkably high recurrence rate (47%) was reported in the work of Hill et al.^22^ Although a radical approach was performed in every patient, nearly all cases of recurrence were seen in patients with incomplete excision. This emphasizes the surgical difficulty of complete excision. No evidence was found on the effect of incomplete excision on recurrence in vascular malformations, compared to complete excision. Hill et al.^22^ found that recurrence occurred rapidly: a mean interval from surgery to recurrence of 10 months was seen. Mendonca et al^6^ observed VMs with bone or joint involvement as a risk factor for recurrence of symptoms after treatment. This observation is based on data of both lower and upper extremity lesions. However, the number of patients in this study is too small to have any statistically significant value. Upton et al found that^2^ both high- and low-flow malformations often required multiple surgeries. The total ratio [number of patients/number of operative procedures] was [141/260] in their study. This ratio was [117/202] in low-flow malformations and [24/58] in high-flow types. These ratios suggest that high-flow malformations require more operative procedures than low-flow malformations.

### Complications

To find a standard treatment for upper extremity vascular malformations, it is essential to not only take the complication rate into account but also the severity of these complications. Skin necrosis, neurological complications and gangrene were considered as most severe complications.

Gangrene was reported in four cases. Hill et al^22^ showed two cases of gangrene, of which one was caused by embolization. Gangrene led to amputation in all four cases. Mendonca et al^6^ believed that sclerotherapy should not be used for distal malformations, as this results in a high risk of terminal ischaemic necrosis. This complication was seen in one patient after sclerotherapy of a VM located in the phalanx. The authors also state that surgical debulking had a high complication rate even though these consisted of minor complications.

Park et al^25^ reported 17 cases of skin necrosis after embolization/sclerotherapy with ethanol. Fourteen of these cases affected small areas and healed with conservative treatment, while two of these patients required surgery for amputation due to gangrene. One of the 17 cases of skin necrosis resulted in autoamputation. Skin necrosis in Park et al^25^ was more common in diffuse malformations than in focal ones (P=0.011). This study also showed that AVMs involving subcutaneous layers had a higher risk to develop skin necrosis after embolization/sclerotherapy than malformations with no involvement of subcutaneous layers (P=0.021). Risk of major haemorrhage during surgery is the main reason for surgeons to stay away from surgical management of congenital vascular malformations, even in cases of presumable reduction of severe symptoms or when patients are suffering from life- or limb-threatening complications.^10^

This review is limited by multiple factors. First of all, not all included studies were of desired methodological quality. Surgical management of upper extremity vascular malformations has been poorly reported over the last decades. Most studies did not report lesion characteristics such as size or what tissues are involved, while these factors could play a critical part in determining surgical outcome. Moreover, most studies included various types of upper extremity vascular malformations did not report outcomes individually for each malformation type.

All articles included in this review were level four case series. These studies were not designed to indicate whether one treatment is superior over another. No cohort-studies or randomized controlled trials have been conducted, comparing surgical and non-surgical treatment of upper limb vascular malformation. As a result of lacking evidence on treatment of vascular malformations of the upper extremity, it would currently not be realistic to write a review based on one particular type . Moreover, each malformation should be assessed individually based on lesion characteristics, e.g. involvement of soft tissue. These characteristics determine what treatment option is suitable, making comparison between different options difficult.

### Recommendations

Current literature provides the following recommendations:

• Upper extremity vascular malformations with mild/intermittent symptoms should be treated conservatively.^6^
• Distal malformations involving tendons/nerves can be treated by surgical debulking, which needs to be executed by those who are experienced in hand surgery.^6^
• Low-flow malformations involving the whole extremity or joints should primarily be treated with sclerotherapy.^6^
Localized, well outlined lesions react well to surgical resection.^6^
Embolization of high-flow lesions should only be performed in patients with significant symptomatology, aiming to eradicate the nidus.^24^

Surgical management could be an effective, safe treatment option. However, current literature describes cases in which surgery results in recurrence or complications. Therefore, non-surgical treatment could be recommended over surgical treatment in these cases. Based on current literature, it is difficult to distinguish these cases, although it seems that large, diffuse lesions tend to have a higher risk of complications and should therefore initially be treated non-surgically. To support this recommendation and identify which factors lead to risk of complications and recurrence, specification of the characteristics of the treated lesions is crucial.

## Conclusion

Surgery for vascular malformations of the upper extremity can be a safe and effective treatment option, although some cases are better off when treated non-surgically. Literature shows various complication rates for non-surgical treatment of upper extremity vascular malformations. To determine in which case surgery is the better option, we should identify factors leading to surgical complications by reporting specific patient and lesion characteristics of the complicated cases.

## Declaration of Competing Interest

None.
